# Molecular regulation of apple and grape ripening: exploring common and distinct transcriptional aspects of representative climacteric and non-climacteric fruits

**DOI:** 10.1093/jxb/erad324

**Published:** 2023-08-17

**Authors:** Sara Zenoni, Stefania Savoi, Nicola Busatto, Giovanni Battista Tornielli, Fabrizio Costa

**Affiliations:** Department of Biotechnology, University of Verona, Strada Le Grazie 15, 37134, Verona, Italy; Department of Agricultural, Forest, and Food Sciences, University of Turin, Largo Paolo Braccini 2, 10095 Grugliasco (Torino), Italy; Research and Innovation Centre, Fondazione Edmund Mach, Via Mach 1, 39098 San Michele all’Adige (Trento), Italy; Department of Biotechnology, University of Verona, Strada Le Grazie 15, 37134, Verona, Italy; Center Agriculture Food Environment (C3A), University of Trento, Via Mach 1, 39098 San Michele all’Adige (Trento), Italy; University of Malaga, Spain

**Keywords:** Apple, climacteric, fruit ripening, grape, hormones, non-climacteric, regulation, transcriptomics

## Abstract

Fleshy fruits of angiosperms are organs specialized for promoting seed dispersal by attracting herbivores and enticing them to consume the organ and the seeds it contains. Ripening can be broadly defined as the processes serving as a plant strategy to make the fleshy fruit appealing to animals, consisting of a coordinated series of changes in color, texture, aroma, and flavor that result from an intricate interplay of genetically and epigenetically programmed events. The ripening of fruits can be categorized into two types: climacteric, which is characterized by a rapid increase in respiration rate typically accompanied by a burst of ethylene production, and non-climacteric, in which this pronounced peak in respiration is absent. Here we review current knowledge of transcriptomic changes taking place in apple (*Malus* × *domestica*, climacteric) and grapevine (*Vitis vinifera*, non-climacteric) fruit during ripening, with the aim of highlighting specific and common hormonal and molecular events governing the process in the two species. With this perspective, we found that specific NAC transcription factor members participate in ripening initiation in grape and are involved in restoring normal physiological ripening progression in impaired fruit ripening in apple. These elements suggest the existence of a common regulatory mechanism operated by NAC transcription factors and auxin in the two species.

## Teaser

This review summarizes the main transcriptional events coordinating the ripening processes in both climacteric (apple) and non-climacteric (grape) models, focusing on transcription factors and hormonal regulation.

## Introduction

Angiosperms often surround their seeds with a fleshy edible structure (Crane *et al.*, 1995), the fruit, that encourages herbivory and thereby promotes seed dispersal and species dissemination (Vasconcelos *et al.*, 2023). Fruits result from the fertilization process following pollination (except in cases of parthenocarpy) and, in fleshy fruit species, the subsequent differentiation of fleshy tissues from the ovary or associated accessory structures due to intense cell division and enlargement. During development and growth, fruits expand by accumulating water in their cell vacuoles, and they synthesize a plethora of environmentally selected metabolites in the fruit skin and flesh while developing and protecting the seeds. Finely orchestrated by hormonal signal and transcription factor activity, these processes collectively constitute fruit ripening (Fenn and Giovannoni, 2021; X. Li *et al*., 2022).

Fruit physiological and molecular processes are nowadays thoroughly investigated on account of the horticultural and economic value of the final products. Fruits are a fundamental element of the daily human diet as suppliers of important nutritional (i.e. carbohydrates) and nutraceutical components (such as vitamins and antioxidant compounds) (Divella *et al.*, 2020). Together with another large array of molecules (contributing for instance to aroma), these contribute to the palatability of a fruit, the appreciation of which results from four main principal quality factors: appearance, flavor (taste/aroma), texture, and the nutritional and nutraceutical components (Costa *et al.*, 2011). These features are derived from the intense kinetics of physiologically coordinated events initiated during fruit formation and development and completed afterward during the ripening phase. The fruit ripening process is a genetically and epigenetically programmed phenomenon, including different processes whose coordination defines the climacteric and non-climacteric types of ripening, mainly distinguished by the progression of respiration and hormonal kinetics. Climacteric fruits, such as apple and tomato, show a rise in respiration at the end of the ripening process accompanied by an increased biosynthesis of ethylene. Non-climacteric fruit, such as grape, citrus, and strawberry, are on the contrary characterized by a continuous decline in the respiration rate and do not require ethylene to complete ripening, although they might respond to this hormone (Giovannoni, 2001).

Understanding the gene network involved in the fruit development and ripening processes is of paramount importance for enhancing fruit quality and security, and especially for the reduction of fruit wastage. Besides conferring valuable quality-related attributes to fruit, the changes occurring during ripening can also lead to the development of negative and undesirable phenomena. Ripening is in fact the last step of the life cycle of the fruit before senescence, and therefore in this phase fruits become more susceptible to various diseases and disorders. To prevent this, fruit can be subjected to several physical or chemical processes to control ripening, mainly through hormonal manipulation. Although this control can contribute to reducing fruit loss or waste, it might not promote the sustainability of fruit production due to increased costs. To this end, the scientific community has employed several strategies to reveal the genetic regulation of these mechanisms, aiming at a better understanding of the physiological machinery governing fruit ripening, from identifying major loci controlling these aspects to analysing gene expression.

The development of microarray platforms (cDNA or oligos) and subsequent RNA-Seq has allowed transcriptome analysis in several fruit crop species, elucidating the transcriptomic regulation occurring in important physiological phases, including fruit ripening. From the initial and pivotal work that shed light on the genetic control of fruit ripening in tomato (Giovannoni, 2004), the affordability of these techniques has allowed the comprehensive investigation of the regulation of these processes also in other species, unravelling the control of ripening in both climacteric and non-climacteric fruits (Lelièvre *et al.*, 1997; Gapper *et al.*, 2013).

In this review, we focus on the transcriptome analysis of fruit formation/development and ripening in two agriculturally relevant species, apple (climacteric) and grapevine (non-climacteric), shedding light on distinct but also common regulatory processes. The specific regulation of these two types of ripening is in fact still to be fully elucidated.

## Development, maturation, and ripening of fruit in apple and grapevine

**Fig. 1.**
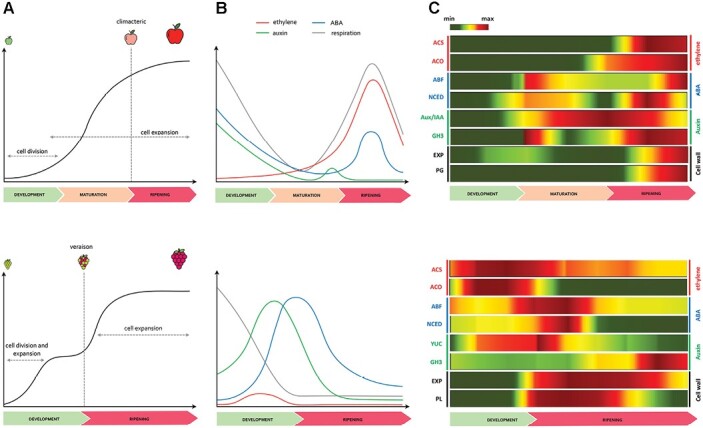
Overview of the two distinct fruit development and ripening patterns characteristic for *Malus domestica* (apple) and *Vitis vinifera* (grape). (A) Growth curve model for both apple (upper panel) and grape (lower panel), with apple displaying a regular single sigmoid curve and grape showing a double sigmoid curve, with two rapid growth phases separated by a lag phase. (B) Profiles of the most important hormones associated with fruit development and ripening in apple (upper panel) and grape (lower panel), including ethylene (red), auxin (green) and ABA (blue) with the respiration rate marked in gray. (C) Heatmaps depicting the relative expression level of representative candidate genes involved in the regulation of the development, maturation, and ripening stages in both apple (upper panel) and grape (lower panel). *ABF*: *ABA responsive element binding factor*; *ACO*: *1-aminocyclopropane-1-carboxylate oxidase*; *ACS*: *1-aminocyclopropane-1-carboxylate synthase*; *Aux/IAA*: *Aux/IAA* genes; *EXP*: *expansin*; *GH3*: *Gretchen-Hagen 3*; *NCED*: *9-cis-epoxycarotenoid dioxygenase*; *PG*: *polygalacturonase*; *PL*: *pectate lyase*; *YUC*: *YUCCA* genes. The color pattern indicates the expression level with green and red for low and high values, respectively. The original data were retrieved from Tadiello *et al.* (2016) for apple, and Fasoli *et al.* (2018) for grape.

In apple (*Malus* × *domestica* Borkh), the main edible fleshy part of the fruit originates from the receptacle (thalamus). Its initial development follows a typical sigmoidal growth, in which the first exponential phase is related to cytokinesis (cell division), while the second phase is more dependent on cytodieresis, guided by a cell enlargement process (Fig. 1A). The fruit ripening phase that follows is characterized by typical climacteric behavior (Fig. 1B), with a late increase in respiration generally coinciding with a rise in ethylene production (Ecker, 1995; Kieber, 1997; Johnson and Ecker, 1998; Pech *et al.*, 2013). This stimulated production triggers the onset of different ethylene-related processes, amongst which the most evident and relevant is the loss of firmness due to expression of several ethylene-related cell wall degrading enzymes. Although most physiological processes contributing to fruit quality are ethylene dependent, ethylene-independent events have also been recently described in apple, such as the hydrolysis of starch into sugars (Doerflinger *et al.*, 2015). Moreover, varieties with a less pronounced ethylene dependency in their ripening have likewise been identified, such as ‘Fuji’, in which the ripening process occurs without a clear peak in ethylene production due to a retroposon insertion in the promoter of *MdACS1* (Supplementary Table S1 for gene full names), the major gene involved in climacteric system 2 ethylene production, which is the auto-stimulatory phase of ethylene production typical of the fruit in full ripening stage (Sunako *et al.*, 1999; Barry *et al.*, 2000; Harada *et al.*, 2000; Costa *et al.*, 2005). The ongoing physiological processes during ripening can be controlled nowadays through different strategies to slow down ethylene production, preventing fruit decay and guaranteeing continuous availability of fresh fruit on the market. The biogenesis of ethylene has already been exhaustively dissected in tomato, the reference model species considered when investigating fruit ripening mechanisms in the pre-genomic era (Vrebalov *et al.*, 2002; Giovannoni, 2004). The gap existing with other species has since been filled thanks to the improvement in sequencing technologies that have made genome sequencing affordable for other crops (Peace *et al.*, 2019). This has enabled genome-wide characterization of the transcriptome and therefore allowed transcriptional elucidation of ripening mechanisms also for apple.

In grapevine (*Vitis vinifera* L.) the fruit undergoes a developmental process described by a double sigmoidal curve characterized by two distinct growth phases (Fig. 1A). During the first phase, the growth of the pericarp is primarily due to fast cell division and enlargement, as well as synthesis and accumulation of organic acids, proanthocyanidins, and other polyphenols, while the second phase of growth requires an increase in cell volume and softening of tissues (Coombe, 1992). The onset of ripening, known as véraison, coincides with seed maturity, and is marked by metabolic changes that enhance the fruit’s appeal and edibility (Conde *et al.*, 2007). Grapevine berry is a typical non-climacteric fruit, in which several modifications are governed mainly by abscisic acid (ABA), which starts to accumulate 2 weeks before véraison and peaks when berries enter the second growth phase (Fig. 1B) (Agudelo-Romero *et al.*, 2013; Castellarin *et al.*, 2016). Although the grapevine berry does not require ethylene to ripen, a slight peak of accumulation of this hormone has been observed just prior to véraison, suggestive of a role for ethylene also in non-climacteric fruit (Tesniere *et al.*, 2004; Böttcher *et al.*, 2013). Instead, low auxin levels are apparently needed at véraison; otherwise, the ripening process might be delayed. In fact, auxin concentration is usually higher during berry formation, subsequently decreasing during berry ripening when this hormone is inactivated by conjugation with amino acids (Böttcher *et al.*, 2010; Fortes *et al.*, 2015). Grapes treated with ethylene, auxin, or inhibitors of ethylene synthesis/perception revealed a biphasic physiological response by the grape berry dependent on the timing of application. Ethylene and auxin can in fact delay ripening when applied early during the pre-véraison phase, and advance ripening (ethylene) or have no effect (auxin) when applied nearer to the time of véraison. These observations depict quite a sharp transition of the grape berry from vegetative to ripening phase. The comparison of expression patterns of eight candidate genes playing key roles in ripening-related hormonal metabolism (*ACS* and *ACO* for ethylene in both species; *Aux/IAA* and *GH3* for auxin in apple; *YUC* and *GH3* for auxin in grape; *ABF* and *NCED* for ABA in both species) and cell-wall dismantling processes (*EXP* and *PG* in apple; *EXP* and *PL* in grape) reflects the different ripening dynamics of the two species, with each element expressed in a crucial step of the process (gene full names are given in Supplementary Table S1). In apple, the expression of these genes is shifted towards the end of ripening, coinciding with the boost of the hormone ethylene, whereas in grape this activation occurs at an earlier stage, mostly during véraison at the transition between berry development and ripening (Fig. 1C). The genes involved in ABA metabolism were expressed during early maturation and early ripening in apple and grapevine, respectively. Those involved in the biosynthesis of ethylene showed a distinct pattern, being expressed much earlier in grapevine than in apple, where they were more expressed at the end of the ripening, coinciding with the ethylene production. The expression profile of genes involved in auxin regulation was more consistent between the two species, while the cell wall modifying genes showed a distinct profile. While in apple the expression of *EXP* was, for instance, induced in a late ripening phase, the expression of *EXP* in grape was more pronounced at the initial stage of ripening. Even considering the above-described differences, mainly related to the different ripening physiology and timing of the two species, this transcriptional behavior clearly highlights a common involvement of the same set of core ripening-related genes, activated in a specific time frame.

## Transcriptional regulation of fruit ripening in apple

### Fruit development and maturation

The molecular regulation of fruit development in apples was initially investigated by employing an early-use cDNA based array characterized by a minimal number of elements (1536), which enabled the identification of 177 genes differentially expressed during fruit development/maturation (Soglio *et al.*, 2009). With a similar tool, it was also observed that the genes identified were mainly involved in primary stress metabolism, defense, amino acid and nucleic acid metabolism, and secondary metabolites (Lee *et al.*, 2007). With a denser oligo type of microarray (15762 oligos identifying 13 000 genes), 1955 differentially expressed genes have been characterized from pollination to maturity in the fruit of the apple cultivar ‘Royal Gala’, further organized in four main clusters: full bloom, early fruit development, mid-development, and ripening (Janssen *et al.*, 2008). This analysis revealed that the fruit metabolic activity decreased from the mid-development stage, as evidenced by the lower number of actively expressed genes associated with metabolism. Moreover two orthologs of Arabidopsis genes were identified, *CDKB* and *CKS1*, with a role in cell cycle progression and mitosis together with specific elements important in fruit growth and development. The rapid progression in sequencing technologies and the release of the apple genome (Velasco *et al.*, 2010) enabled transcriptomic assessment at a genome-wide scale. Investigation of a somatic mutation related to the different accumulation of anthocyanins in apple peel unraveled the differentially expressed genes and methylated regions, especially related to *ANS* and *F3H* genes encoding key enzymes of the red coloration of the peel (Jiang *et al.*, 2019). In the same study, the expression of *MdMYB114*, encoding an MYB type transcription factor necessary for stimulating red coloration in apple peel, was characterized. The accumulation of anthocyanin, investigated through RNA-Seq and a weighted gene co-expression network (WGCNA), identified a module represented by 34 genes associated with the biosynthesis of anthocyanin by comparing a normal red-skin type apple cultivar (‘Gala’) with a derived somatic mutant (‘Blondee’) (El-Sharkawy *et al.*, 2015). The transcription analysis identified specific elements known to be involved in the control of anthocyanins, together with the identification of 22 novel elements, including *MdMYB10* and *MdGST*, that showed down-regulated transcription in the mutant compared to its red-colored parent. In addition, the fruit peel during the development and maturation process can sometimes accumulate suberin in the outer epidermal layer (russeting). The repression of cuticular biosynthetic genes together with a parallel increase in expression of suberin and triterpene-related genes was observed in a bulk-transcriptomic profiling approach in russeted apples, when compared with normal waxy fruit (Legay *et al.*, 2015). This comparison also identified many differentially expressed genes related to fatty acid elongation that were less expressed in affected fruit, suggestive of a reduced accumulation of C16-acyl carrier protein, together with key elements involved in the regulation of cutin and suberin synthesis, such as *GPAT*. On the contrary, the genome-wide transcription survey revealed that in russeted fruit a *CESA*, specific *XETs*, and a *COBRA-like* were instead overexpressed. To better elucidate the mechanism controlling suberin synthesis and deposition, a transcriptional signature of suberin polymer assembly throughout the angiosperms underlined a high level of conservation (Lashbrooke *et al.*, 2016). The transcription comparison across species featured putative orthologs particularly enriched in suberified tissues of both apple and tomato, and belonging to pathways involved in aromatic compounds, phenylpropanoids (*4CL5*, *FAH1*, and *CCoAOMT*), suberin (*ASFT*, *GPATS*, *CYP86B1*, *ABCG20*, and *LTPG5*), fatty acids (*FATB*), and lignin. In a more comprehensive co-expression analysis, including the transcriptomic dataset of other species, such as grapevine, potato, different tomato tissues, and rice, the two transcription factors *MYB107* and *MYB9* were identified, and transgenic Arabidopsis lines *myb107-myb9* displayed seeds with decreased suberin polymerization when compared with the wild-type ‘Columbia’ ecotype. The transcriptomic analysis integrated with a more comprehensive multi-omics approach was also adopted to define valuable biomarkers suitable for predicting an optimal harvest, ensuring therefore the best quality features (Favre *et al.*, 2022). Among the most enriched annotation clusters, one of the most important biomarkers was represented by *MdPG1*, a gene reputed to encode a cell wall modifying protein that depolymerizes demethylesterified homogalacturonan and whose expression generally increased towards the late harvesting period. The transcript profile of this gene, together with the two main elements involved in ethylene metabolism (*MdACS1* and *MdACO1*), permitted the informative monitoring of the principal processes characterizing apple maturity and ripening, defining therefore the most suitable harvesting period to improve quality, especially considering the final commercial destination of the fruit (Dilley *et al*., 1995; Busatto *et al.*, 2016).

Recent work has also highlighted that beside transcriptional variation, fruit maturation and ripening in apple can be also controlled by epigenetic regulation. Hu *et al.* (2022) have in fact reported that histone deacetylase 19 (MdHDA19) can play a role in the control of the ethylene biosynthesis. MdHDA19, promoting H3k9 deacetylation, can form a MdERF4–dTPL4 complex repressing the expression of *MdACS3a* gene, specifically active in the early maturation phase. MdHDA19 was furthermore found to interact with MdMADS6, which in turn promoted carotenoid accumulation in apple fruit (Q. Li *et al*., 2022).

### Climacteric ripening and postharvest transcriptional control

Apple is distinguished by a climacteric type of ripening, where myriad of chemical and physical modifications occurring in the fruit are generally coordinated by a burst of ethylene production by system 2 (Barry *et al.*, 2000) associated to the respiration peak. In apple, the increase in ethylene occurs during the last phase of ripening, taking place normally after harvest, due to its horticultural maturity. The late coordination of gene expression in this phase, compared with grape, is illustrated in Fig. 1C, in which the time of activation is depicted by a core set of elements specifically involved in the coordination of fruit ripening in apple. Considering the impact of ethylene in coordinating the fruit ripening processes in apples, the majority of transcriptomic investigations focused on the late phase of ripening, comparing the normal and distorted physiology provoked by the application of 1-methylcyclopropene (1-MCP). This molecule, interfering with ethylene receptors, extends the storability of fruit and enables the deciphering of several ethylene-related processes. In fact, 1-MCP application is able to impair ethylene-related gene expression, down-regulating the transcript level of elements involved in ethylene biosynthesis and signaling, such as *SAM*, *ACS*, *ACO*, *EIL*, *EREBP*, and *ERF3* (Costa *et al.*, 2010). It is also worth noting that the application of the ethylene competitor induced the expression of a set of genes related to auxin (*IAA7*), pyruvate decarboxylase, and ripening-related proteins. The production of ethylene in apple coordinates several ripening-related processes, in particular the dismantling of the cell wall structure, triggering the expression of genes such as *XET*, *xyloglucan endo 1-4-glucanase* (*XEG*), and *PG*, which were in turn repressed by 1-MCP. The expression of the apple polygalacturonase *MdPG1* is dependent on the level of the hormone ethylene and the action of MdMAPK3. The kinase activity of MdMAPK3 is stimulated by ethylene and guides the phosphorylation of MdNAC72, a repressor of *MdPG1.* The ubiquitination and subsequent degradation of MdNAC72 by MdMAPK3 enhances the expression of this hydrolase, promoting fruit softening (Wei *et al.*, 2023). The use of a whole genome-based oligo array for apple (Tadiello *et al.*, 2016) also elucidated that 1-MCP treatment, beside repressing important climacteric-dependent and ripening-related genes, also stimulated specific elements involved in the transcriptional circuit regulating fruit ripening and shed light on a particular hormonal cross-talk between ethylene and auxin during the postharvest phase. It is also worth noting that among the 38.2% of genes potentially regulated by the application of 1-MCP, the majority were represented by transcription factors (*NAC* and *MADS*) and elements involved in the signaling mechanism of both hormones, such as *AUX/IAA* and *ERF*. Among the category of transcription factors, the group that restored significant activation following the application of 1-MCP was represented by NAC, a class of transcription factors known to be involved in several biological processes. In tomato, *NAC1* was in fact induced by auxin, suggesting an important regulatory role in the control of ripening (Meng *et al.*, 2016). In this scenario, the impaired ripening can induce the fruit to de-repress the auxin machinery towards a re-activation of the hormonal pathway, consistent with the observation that auxin can also promote the initiation of ethylene metabolism (Morgan and Hall, 1962; Trainotti *et al.*, 2007; Busatto *et al.*, 2016).

The hormonal cross-talk was further validated through metabolite analysis and an RNA-Seq approach, finding that during the postharvest phase in fruit with a delayed ripening physiology, an actual increase of auxin was observed in three apple cultivars: ‘Golden Delicious’, ‘Granny Smith’, and ‘Fuji’ (Busatto *et al.*, 2021). Genome-wide transcription analysis and KEGG pathway database interrogation revealed that most of the genes were associated with plant hormone and signal transduction pathways, being mainly related to ethylene and auxin. The interplay between these two hormones was moreover confirmed by the contrasting expression of *GH3* and *ILL* genes, with a role in the maintenance of auxin homeostasis. In fact, although both elements are involved with the metabolism and regulation of auxin, the expression profile of *GH3* (involved in the auxin-conjugation process) (Chapman and Estelle, 2009; Ljung, 2013; Kramer and Ackelsberg, 2015; Sanchez Carranza *et al.*, 2016) was more correlated with the transcription profile of ethylene-related genes, while *ILL* (involved in regulation of the de-conjugation process) was instead more consistent with the pattern of auxin-associated genes.

The application of 1-MCP to retard fruit ripening was shown to have a crucial impact also on the production of important volatiles producing the fruit aroma in apple (Tadiello *et al.*, 2016; Busatto *et al.*, 2018). To this end, Schaffer *et al.* (2007), investigating the transcriptome and metabolic regulation of aroma production in a transgenic line of ‘Royal Gala’ apple cultivar silenced for *MdACO1*, observed that the biosynthesis of ethylene consistently reduced the production of aroma, which could be effectively restored (enhanced 12-fold) by exogenous application of ethylene. This functional investigation revealed that specific categories of volatile organic compounds, such as esters, were ethylene dependent, while others, such as aldehyde and alcohols, were only partially regulated by this hormone.

In addition to 1-MCP, the extension of storage can also be promoted by the application of low temperature and low oxygen, which can also stimulate the development of serious disorders as a result of fermentation processes and hypoxia. Fruit stored in low oxygen conditions displayed a higher accumulation of specific metabolites, in particular ethanol and alanine, and an initiation of a low oxygen acclimation process guided by elements belonging to the ERF-VII group (Cukrov *et al.*, 2016). This finding suggests that the oxygen sensing system based on the N-end rule pathway originally identified in Arabidopsis or in submerged rice (Licausi *et al.*, 2011) is possibly conserved across species and activated also in apple stored in a modified controlled atmosphere.

Due to the importance of postharvest storage in the preservation of quality attributes of the fruit reached at the time of harvest, transcription analysis was also employed to elucidate the onset of superficial scald, one of the most important post-harvest disorders in apple. The genome-wide transcription profiling carried out comparing scalded symptomatic fruit (showing a dark area on the peel) and asymptomatic fruit in which the disorder was prevented through the application of 1-MCP elucidated that the protection of the fruit by this metabolic phenomenon was due to synergistically coordinated simultaneous events. The RNA-Seq profile reveals that the putative mechanism was related to direct control of *MdPPO*, a polyphenol oxidase gene expressed with an ethylene-dependent pattern encoding a key enzyme involved in oxidation of chlorogenic acid into quinone and melanin (Amiot *et al.*, 1993). In this investigation, it was also observed that a core set of differentially expressed genes was positively regulated by the application of 1-MCP. Within this group, one gene was distinguished for its high level of expression and represented by an *MdS6PDH*, whose expression was consistent with the metabolic accumulation of the sugar alcohol sorbitol. The actual role of this polyol in promoting resistance to low temperature was further validated with a functional heterologous approach in Arabidopsis. The generation of an *MdS6PDH* overexpressing line led to an accumulation of sorbitol, which conferred a cold-resistance phenotype compared with the wild type (Busatto *et al.*, 2018). Karagiannis *et al.* (2020) established a transcriptional network for this aspect, identifying several transcription factors co-regulating with different genes and defining a distinct transcriptional signature related to the application of 1-MCP and the prevention of superficial scald development. In this work an *HD-Zip* gene was identified and co-regulated with the expression pattern of *PPO*, a gene playing a central role in the control of this oxidative disorder.

## Transcriptional regulation of fruit ripening in grapevine

### Fruit development and ripening

Numerous studies to examine the molecular aspects of fruit development in grapevine have been conducted over the past two decades and are extensively reviewed in Zenoni *et al.* (2021). These investigations have identified links among molecular modifications and biological processes involved in berry development and ripening, such as hormone biosynthesis, regulation and signaling, cell wall metabolism, and responses to both abiotic and biotic stimuli. By studying the transcriptome at various stages of berry development, a significant rearrangement in gene expression when transitioning from vegetative to ripening phases was underlined. This rearrangement was mainly distinguished by a down-regulation of genes rather than up-regulation (Fasoli *et al.*, 2012), confirmed by the transcriptome analysis of 10 grapevine varieties showing that the number of genes expressed in the vegetative phase decreased significantly during ripening (Massonnet *et al.*, 2017). This reduction was specifically noticeable among genes with medium to high expression levels. Furthermore, the most significant transcriptional modulation occurred during transition from pre- to post-véraison stages. A group of genes displaying consistent expression patterns during formation and ripening phases across varieties was discovered, thus representing shared core transcriptomic changes occurring during the process. These changes were categorized into six expression clusters, out of which three encompassed genes peaking in expression during the formation phase, while the remaining clusters contained genes showing maximum expression during the ripening phase. Additionally, a higher degree of diversity was noted at the gene expression level among red berry cultivars when compared with white, indicating that the regulation of numerous biological processes is strongly influenced by anthocyanin biosynthesis (Massonnet *et al.*, 2017). Fasoli *et al*. (2018) discovered that the primary changes in gene expression surmised to govern berry development remained constant across different years. These changes were manifested through patterns of harmonized waves of gene expression during the stages of berry formation, véraison/mid-ripening, and late ripening (Zenoni *et al.*, 2021).

The multifaceted physiological processes occurring during berry development could be meaningfully depicted by functional annotation of genes that mostly fell into three waves of expression. During early berry development/pre-véraison, a gene expression pattern emerged (first wave) involving genes that were initially highly expressed and down-regulated thereafter (Fasoli *et al.*, 2018). Most of these transcripts were associated with types of energy metabolism, such as those involved in oxidative phosphorylation and carbon fixation. The phase of berry formation before véraison was characterized by the expression of photosynthesis-related genes in the berry, which were progressively down-regulated as development continued. During this phase, various genes associated with glycolysis, as well as starch and sucrose metabolism and glyoxylate and dicarboxylate metabolism, were also observed to be expressed (Dokoozlian, 2000). Genes related to cell division and auxin signaling, such as those for ARFs, efflux carriers, and auxin-induced proteins were also identified, indicating the crucial role of this hormone in regulating cell division in the early phase of berry formation. Various homeobox genes, bHLH proteins, and zinc finger proteins were identified among the transcription factors. Many genes expressed in this vegetative wave were part of the processing network categories, comprising ribosomal proteins and genes engaged in RNA metabolism. Finally, numerous transport-related genes were observed, such as those for ABC transporters, MATE efflux proteins, nodulins, and sugar transporters.

During the onset of ripening, another gene expression pattern was observed (the second wave), involving genes showing a maximum expression around véraison, followed thereafter by a subsequent decline (Fasoli *et al.*, 2018). Most genes following the second wave of expression pattern can be ascribed to the various ripening events and metabolisms, such as those encoding sugar transporters that likely facilitate apoplastic phloem unloading during ripening, a crucial process addressing high osmotic potential and hydrostatic pressure in the berry vacuole. Genes for sugar transporters, such as HT6 and SWEET10, tonoplastic and plasma-membrane aquaporins, and cell wall expansins are then sharply repressed at the phloem arrest (corresponding to the berry maximal volume expansion) (Savoi *et al.*, 2021). Moreover, some genes within this expression trend may contribute to the sugar-sensing that acts as a trigger to control the onset of berry ripening (Gambetta *et al.*, 2010). The second wave of gene expression also included transcription factors such as the R2R3-MYB *VviMYBA1* regulating anthocyanins (Walker *et al.*, 2007), basic leucine zipper transcription factors, and other zinc fingers. Lastly, numerous genes involved in phenylpropanoid and flavonoid/anthocyanin pathways were noted, like *LDOX* and *AOMTs*. Notably, several genes of this second wave of expression showed evidence of a differential modulation at véraison (i.e. they were up-regulated in one variety and not in the other and vice versa) related to varietal traits (Fasoli *et al.*, 2018). For instance, in ‘Pinot Noir’, known for having a higher abundance of stilbenes compared with ‘Cabernet Sauvignon’, the well-known regulators *VviMYB14* and *VviMYB15* and several stilbene synthase genes (*STSs*), controlling the expression of stilbenes (Vannozzi *et al.*, 2012; Höll *et al.*, 2013), followed the second wave expression pattern during berry development and exhibited little expression in ‘Cabernet Sauvignon’. Stilbenes are a class of phenylpropanoid compounds constitutively expressed in healthy grape from the onset of ripening and enhanced upon biotic and abiotic stresses with a function in defense strategies. In line, the *Vvi3AT* gene, which is accountable for the acylation of anthocyanin (Rinaldo *et al.*, 2015), showed a peak of expression around véraison in ‘Cabernet Sauvignon’. On the contrary, this gene was not expressed in ‘Pinot Noir’, which at the same time has no acylated anthocyanins in the skin. Acylation confers stability to the anthocyanin molecule and a higher content of acylated anthocyanin is considered a valuable enological trait in wine grape cultivars. The discovery of genes exclusively exhibiting a peak of expression around véraison in either ‘Pinot Noir’ or ‘Cabernet Sauvignon’ provides an explanation for the distinct traits characterizing each variety. Overall, genes that exhibited the second wave of expression during berry development are thought to play a crucial role in initiating the ripening phase.

During the late stages of berry ripening, a final gene expression pattern developed (the third wave) consisting of genes with their expression increasing gradually from véraison onwards (Fasoli *et al.*, 2018). Remarkably, these genes mainly encoded transcription factors and components of the genetic information processing network, such as those involved in cellular component organization, nucleic acid processing, and other processes contributing to significant transcriptomic changes during berry maturation. Genes related to cell wall metabolism, carbohydrate metabolism, hormone stimulus (particularly ABA and brassinosteroids) and stress response, such as the defense-related R proteins, were also part of the third wave of gene expression. The *UFGT* gene, which represents a fundamental step in anthocyanin biosynthesis, was also present, along with several structural genes of the phenylpropanoid/flavonoid pathway. Interestingly, the third wave of gene expression appeared to be correlated with the rate of berry ripening, with a steeper slope observed in the early-ripening variety ‘Pinot Noir’ compared with the late-ripening ‘Cabernet Sauvignon’.

By defining the three waves of gene expression during berry development, researchers have gained unique insight into the different phases of this process. This has provided the basis for identifying the master regulators that trigger onset of ripening. Using advanced bioinformatic tools, a small set of genes named switch genes has been identified and proposed as key regulators in controlling the transition from vegetative to ripe berry (Palumbo *et al.*, 2014; Massonnet *et al.*, 2017). The switch genes showed minimal expression during the vegetative phase but a significant increase at the onset of ripening, following the second expression pattern (Supplementary Table S2) (Fasoli *et al*., 2012). These genes were also found to be inversely correlated with elements associated with the first wave of expression, which were down-regulated during the developmental shift. These observations suggest these switches are critical genes in regulating the immature to mature phase transition during grape berry development, possibly by suppressing vegetative pathways and activating ripening-related processes. Further investigation was carried out by examining the temporal window of initial transcriptional events that trigger the onset of ripening (Fasoli *et al.*, 2018). The importance of the switch genes has also been demonstrated in the analysis of grape bunches affected by berry shrivel, a physiological ripening disorder. In such cases, many switch genes did not ‘switch’ at vérasion, preventing those bunches from properly entering and completing the ripening program, remaining effectively immature (Savoi *et al.*, 2019).

### Post-ripening molecular events

Grapes are typically harvested at the commercial ripening stage for fresh consumption or wine production, when the major ripening parameters meet ideal organoleptic or oenological requirements.

In order to maintain berry quality during postharvest storage of table grapes, different technologies are employed but little is known about the molecular events taking place during postharvest. Few transcriptomic studies are present in literature, as most of the analyses focused on the expression of a small number of genes responding to the different postharvest treatments (reviewed by Romero *et al.*, 2020). For example, Maoz *et al.* (2019) demonstrated that low temperature but not high CO_2_ storage condition induced stilbene biosynthesis by the activation of the stilbene regulator gene *VviMYB14*.

On the contrary, regarding wine grapes, a number of transcriptomic studies describing the molecular dynamics characterizing the post-harvest period have been published. During this phase, which is known as withering, the berries develop unique quality traits, normally absent at the time of harvest (Costantini *et al.*, 2006; Degu *et al.*, 2021). At the molecular level, it was initially discovered that expression of the *STS* gene was induced after grape harvest (Versari *et al.*, 2001). Subsequent transcriptomic studies (Zamboni *et al.*, 2008; Rizzini *et al.*, 2009; Fasoli *et al.*, 2012) revealed that changes in gene expression involved transcripts associated with multiple biological processes, which may continue to occur several weeks after harvesting. These findings suggest that most of the metabolic changes observed in dehydrating grapes were regulated primarily at the transcriptional level, as part of an over-ripening/senescence program, or related to detachment from the plant and dehydration stress.

An in-depth examination of the metabolite and transcript profile changes occurring after harvest in six different red berry genotypes revealed that secondary metabolism was particularly affected, with a strong activation of genes related to stilbene, terpene, and lignin metabolism, and a general repression of genes involved in anthocyanin biosynthesis (Zenoni *et al.*, 2016). Additionally, it was noted that laccase-encoding genes were widely induced in most genotypes. Laccases have been reported to facilitate the oxidation and polymerization of phenolic compounds, and widespread expression of these genes might be linked to the formation of viniferins, which are dimeric and oligomeric stilbenes that accumulate in dehydrated berry skin (Pilati *et al.*, 2021).

Even though grapevine fruits are non-climacteric, several transcriptomic studies focusing on the grape post-ripening phases have reported the induction of several genes related to ethylene, such as *SAM* synthase and *ACO* (Zamboni *et al.*, 2008; Rizzini *et al.*, 2009; Zenoni *et al.*, 2016). These findings, along with the effects of ethylene treatment on berry metabolism observed in other studies (Botondi *et al.*, 2011; Becatti *et al.*, 2014), suggest that postharvest metabolome and transcriptome rearrangement may be partly regulated by ethylene. Additionally, because some auxin-related genes are also modulated, a cross-talk between auxin and ethylene in response to water loss stress caused by postharvest dehydration can be hypothesized (Tonutti and Bonghi, 2013). The post-harvest withering induced changes in the level of transcripts related to cell-wall modification, consistent with the observed rearrangements of cuticle components, cellulose, xyloglucans, and pectins (Zoccatelli *et al.*, 2013; Fasoli *et al.*, 2019). Moreover, many transcription factors were found to be differentially expressed, with many belonging to families such as WRKY, MYB, bHLH, NAC, and Zinc finger C3HC4-type (Zenoni *et al.*, 2016). Although the specific function of most of these transcription factors is still unknown, they may play a critical role in signaling pathways involved in the dehydration stress response or in the regulation of late berry developmental processes such as senescence (Gómez *et al.*, 2014).

## Common transcriptional regulation between climacteric and non-climacteric fruit from the apple and grape berry perspective

The two types of ripening physiology, climacteric and non-climacteric, are undoubtedly distinguished by specific phenomena, although the existence of common regulatory mechanisms has been presented (Cherian *et al.*, 2014). Several lines of evidence suggest the existence of a hormonal signal interplay possibly coordinating the ripening of both climacteric and non-climacteric fruits. The conservative transcription regulation of ripening was initially implied by the finding that a homolog of MADS-RIN, a major transcription factor regulating the ethylene-dependent ripening processes in tomato, was also expressed in non-climacteric fruits, such as strawberry and pepper (Vrebalov *et al.*, 2002; Adams-Phillips *et al.*, 2004; Barry and Giovannoni, 2007; Lee *et al.*, 2010). Comparative genomics carried out between tomato and pepper also revealed a similar transcription pattern of genes for fruit ripening master regulators, such as *NOR*, *TAGL1*, and *RIN*, during fruit development and suggested a conservative mechanism between these two categories (Fuentes *et al.*, 2019). It has been also recently discovered that ABA, a typical hormone regulating ripening in non-climacteric fruits (Fan *et al.*, 2022), seems to control lycopene accumulation in tomato, a typical ethylene-dependent event (Zhang *et al.*, 2009; McAtee *et al.*, 2013; Peace *et al.*, 2019). The overexpression of *Lycopene β-Cyclase* (*LCYb*) induced, in fact, an accumulation of ABA and a decreased production of ethylene (Diretto *et al.*, 2020).

Concerning the two main species included in this review, a small peak of ethylene and a rise in expression of ethylene-related genes (*EIL-like*, *ETR*, and *AP2/ERF*) have been observed in grapevine berry (Tesniere *et al.*, 2004; Licausi *et al.*, 2010) as well as in other non-climacteric species, such as strawberry (Trainotti *et al.*, 2005) and pepper (Lee *et al.*, 2010). Exogenous treatment with ethylene or 1-MCP in grapevine either stimulated or repressed the expression of ripening-related genes, similarly to what occurs in climacteric species (Paul *et al.*, 2012). Recently, it was found that ethylene treatment promotes grape ripening and that VviERF75 may regulate fruit ripening by promoting ethylene biosynthesis and chlorophyll degradation (Li *et al*., 2023a). Likewise, an increase of ABA concentration during apple ripening has also been shown, preceding the rise of climacteric ethylene accumulation (Vendrell and Buesa, 1989). Moreover, ABA seems to be essential for the initiation and reinforcement of the climacteric ethylene production loop in apple after harvest and cold storage (Fernández-Cancelo *et al.*, 2022). The application of ABA was found to accelerate the production of short-chain esters (such as hexyl propionate and ethyl-2-methyl butyrate) and enhance the expression of volatile organic compound biosynthetic genes (such as *AAT2*) during the ripening of ‘Orin’ apples. ABA treatment also stimulated ethylene production and led to shifts in ethylene peaks that corresponded to the expression of ethylene synthesis genes (such as *ACS1/3* and A*CO1*), implying that ABA may act synergistically with ethylene as a positive regulator during the ripening stage. Furthermore, the endogenous levels and expression of an ABA biosynthesis gene (*NCED1*) and a signal transduction gene (*ABF2-like)* increased as ripening progressed (Wang *et al*., 2018). *NCED* was highly expressed during the onset of ripening in grapevine as well as in peach (Chernys and Zeevaart, 2000; Zhang *et al.*, 2009; Degu *et al.*, 2014). In pepper, ABA promotes capsanthin biosynthesis by the activation of an R-R-type MYB transcription factor gene, *DIVARICATA1*, which could be regulated by the ripening regulator MADS-RIN (Song *et al.*, 2023). The RNA-Seq analysis, carried out before and after ripening in apple, identified genes known to be involved in the pathway of ABA. The transcript level of the *NCED1* gene was highly accumulated in the late ripening stages, suggesting a role in controlling the fruit ripening also in apple (Onik *et al.*, 2018). In addition to ABA, auxin can play a crucial role in coordinating ripening in both species. Through a transcriptional analysis it has been observed that exogenous application of naphthalene acetic acid induced transcription of ethylene related genes in grapevine (Ziliotto *et al.*, 2012). The same interaction between these two hormones has also been recently reported for apple (Busatto *et al.*, 2021), shedding light on the potential role of auxin as a common ripening regulator.

Ripening control in fleshy fruit is highly coordinated by several families of transcription factors that activate or repress the expression of downstream ripening-related elements. Among them the R2R3-MYB family members have been demonstrated to play key roles in the different branches of the phenylpropanoid pathway. In both apple and grape species, phenylpropanoid compounds represent an abundant class of secondary metabolites that influence the color, aroma, and organoleptic characteristics of the ripe fruit (Boyer and Liu, 2004; Conde *et al.*, 2007). In apple, an R2R3-MYB type of transcription factor was related to anthocyanin regulation with the activation of *MdMYB1*, *MdMYB10*, and *MdMYB110a* genes (Takos *et al.*, 2006; Chagné *et al.*, 2007; Fang *et al.*, 2023), whereas MdMYB9, MdMYB11, MdMYB12, MdMYBPA1, and MdMYB308, were shown to promote the accumulation of both proanthocyanidins and anthocyanins (Vimolmangkang *et al.*, 2013; An *et al.*, 2015; Zhang *et al.*, 2022; Li *et al*., 2023b). In grapevine, anthocyanin synthesis in red berry genotypes is under the genetic control of VviMYBA1 and VviMYBA2 (Kobayashi *et al.*, 2004; Walker *et al.*, 2007). VviMYBPA1 and VviMYBPA2, together with VviMYBPAR, coordinate the biosynthesis of proanthocyanidin (Bogs *et al.*, 2007; Terrier *et al.*, 2009; Koyama *et al.*, 2014),   VviMYBF1 controls the synthesis of flavonols (Czemmel *et al.*, 2009), and VviMYB14 and VviMYB15 are involved in the synthesis of stilbenes (Höll *et al.*, 2013; Orduna *et al.*, 2022). The MYB factors VviMYB5a and VviMYB5b, which were previously identified as regulators of the overall flavonoid pathway (Deluc *et al.*, 2006, 2008; Cavallini *et al.*, 2014), play a role in vacuolar acidification, representing another crucial aspect of berry quality (Amato *et al.*, 2019).

Although MYBs represent one of the largest classes of transcription factors within each genome, other categories of transcription factor can also play an important role in the overall control of the fruit ripening processes. Among them it is worth noting members of the NAC family (NAM, ATAF1/2 and CUC2) that have been recently documented as master regulators of fruit ripening in both climacteric and non-climacteric fruits (Forlani *et al.*, 2021; Liu *et al.*, 2022; Liu *et al.*, 2023). Interestingly, it was recently demonstrated in both apple and grapevine that NAC transcription factors can directly regulate MYB-type transcription factors. In apple, the overexpression of *MdNAC52* leads to anthocyanin accumulation, while the induction of *MdMYB9* and *MdMYB11* expression revealed their involvement in proanthocyanidin biosynthesis (Sun *et al.*, 2019). Recently, MdNAC042 was demonstrated to positively regulate anthocyanin content through a dimerization with MdMYB10 (Zhang *et al.*, 2020). In grapevine, overexpression of *VviNAC17* in cultured *in vitro* calli was shown to induce activation of *VviMYB14/VviMYB15* and downstream stilbene-related genes, as well as *VviMYBA1/VviMYBA2* and downstream anthocyanin-related genes (Badim *et al.*, 2022). VviNAC60 was recently found to directly activate expression of *VviMYB14* and together with VviNAC03 significantly activated *VviMYBA1* promoter, increased by the heterodimeric form of these two NACs (D’Inca *et al.*, 2023). NAC members are also involved in the regulation of hormone signaling. The NAC SlNOR, a core transcription factor regulating fruit ripening in tomato (Giovannoni, 2004; Gao *et al.*, 2020), permanently binds the promoter of *SlACS2*, a key element in the biogenesis of ethylene, as well as activating the transcription of *SlAREB1*, encoding a transcription factor downstream of the ABA signaling pathway. The induced expression of *SlNOR* through direct binding of its promoter by SlRIN and SlAREB1 suggests transcriptionally regulated cross-talk between ABA and ethylene, again supporting a possible common regulatory mechanism between climacteric and non-climacteric fruit species (Fujisawa *et al.*, 2013; Mou *et al.*, 2018; Liu *et al.*, 2022). In apple it has been demonstrated that MdNAC1 and MdNAC2 can interact with genes involved in the ethylene transduction system, such as *reversion to ethylene sensitivity* (*MdRTE*). Moreover, during postharvest ripening, the expression of *MdNAC1*, *MdNAC78*, and *MdNAC181* (*MDP0000119446*) was stimulated by the application of 1-MCP and repressed by ethylene (Busatto *et al.*, 2017). In transgenic Arabidopsis overexpressing the grapevine *VviNAC17*, enhanced resistance to drought was observed together with a significant up-regulation of ABA and stress-related genes (Ju *et al.*, 2020). The ectopic expression of *VviNAC26* in tomato stimulated the expression of genes related to both the ethylene and ABA pathways, inducing consequent early ripening leading us to hypothesize the transcriptional control of this transcription factor and a common conserved functional regulator (Zhang *et al.*, 2021). Recently, it was shown that both *VviNAC60* and *VviNAC03* ectopically expressed in the tomato *nor* mutant can restore ethylene production, thus inducing the progression of ripening (D’Inca *et al.*, 2023).

**Fig. 2.**
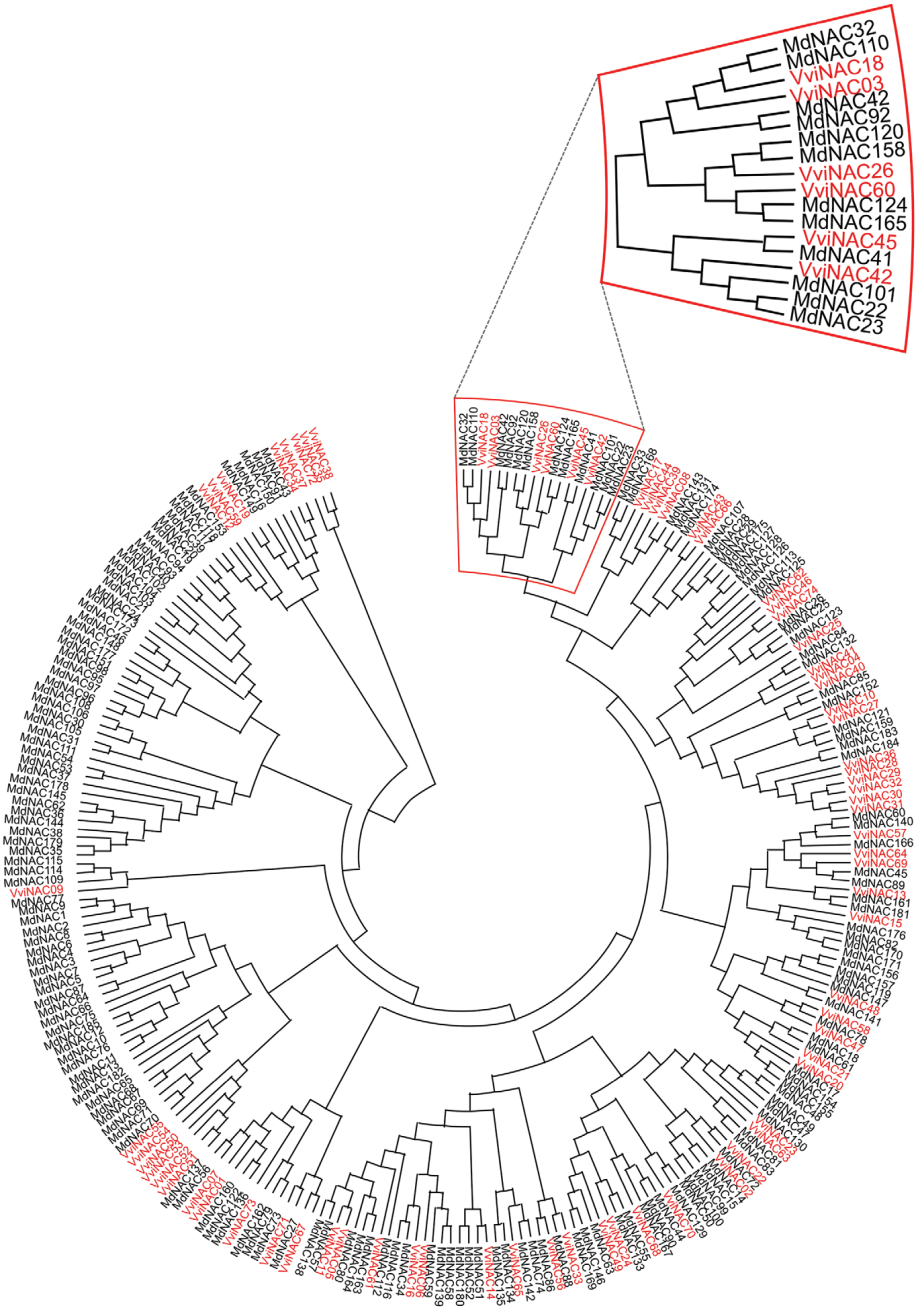
Phylogenetic analysis of apple and grape NAC transcription factors. The 185 NAC protein sequences of apple (black) have been retrieved from Su *et al.* (2013). The 74 NAC protein sequences of grapevine (red) have been retrieved from Wang *et al.* (2013). The sequences were aligned using MUSCLE with default settings. The unrooted phylogenetic tree was constructed in MEGA v11 using the neighbor-joining method based on pairwise deletion and bootstrap analysis with 1000 replicates. The clade to which VviNAC60 belongs has been emphasized.

Altogether, these studies indicate that NAC members could exert similar key roles in apple and grape, and as a step forward in hypothesizing a possible similar and conserved function between the two species, a phylogenic tree including 185 apple and 74 grapevine NAC members has been constructed (Fig. 2). The analysis of the sequence similarity revealed that VviNAC60, recently demonstrated to belong to the tomato NOR clade (D’Inca *et al.*, 2023), was grouped in the same clade with two NACs of apple, MdNAC124 and MdNAC165, both included in the Whole-Genome Apple Array (WGAA) specifically designed to comprehensively investigate the transcriptional reprogramming occurring during the late phase of apple ripening (Tadiello *et al.*, 2016). *MdNAC165* (corresponding to the ID MDP0000404409 or the WGAA ID MD_17_12849) expression was stimulated about 2-fold by treatment with 1-MCP compared with normal ripening, suggesting a role as an early regulator of the fruit maturation process; it can also be de-repressed in the case of a disturbed scenario leading to the re-establishment of a physiological ripening progression. The phylogenetic tree revealed other MdNAC members were very closely positioned to VviNAC60 in the same clade. By looking at their expression trend during fruit ripening we found a similar expression pattern to VviNAC60, in particular for MdNAC32 and MdNAC110 (Supplementary Fig. S1). Due to their expression profile and similarity to VviNAC60, further analysis should be carried out to establish if these NACs share an ancestral function coordinated by auxin. This hormone is in fact the principal signal coordinating both the non-climacteric ripening in grape and the initiation of climacteric ripening in apple, together with restoring normal conditions in the case of ethylene interference.

In fruit-omics investigation it has been widely debated which trigger initiates and orchestrates the ripening process. To better elucidate ripening control in diverse fruits, a genome encode analysis was carried out to disclose the molecular circuit deputed to control ripening (Lü *et al.*, 2018). By integrating a multidimensional approach and employing different genome-wide transcription together with DNA methylation histone modification analysis, three transcriptional circuits were identified. In eudicots with recent whole genome duplication (WGD) the climacteric feedback circuit involves MADS-box transcription factors, while in species that did not experience a recent WGD, the ethylene regulation is operated through NAC transcription factors. In the hybrid version, first a NAC-type feedback circuit is present with an additional loop between NACs and MADS for a double control over ethylene production when the first loop is blocked by the presence of the ethylene inhibitor 1-MCP after ripening initiation. Interestingly, in all cases these transcription factors showed EIN3 motif binding sites in the promoter region of their genes, inducing expression of *ACS* and *ACO* ethylene biosynthetic genes (Alexander and Grierson, 2002).

**Fig. 3.**
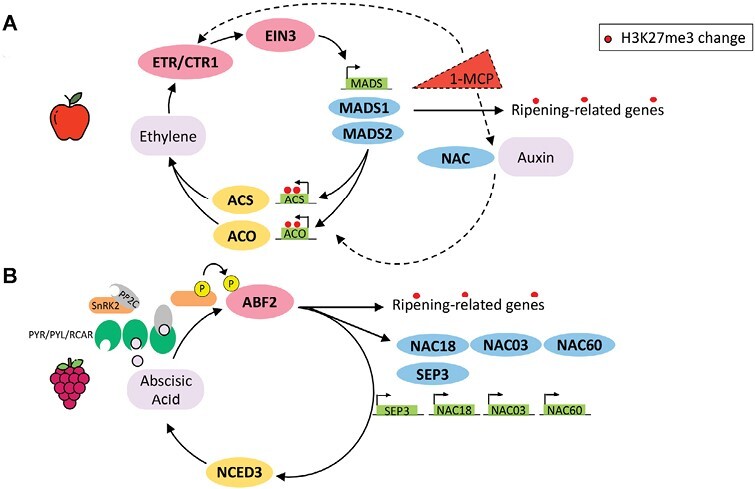
Ripening regulation models in apple and grape controlling climacteric and non-climacteric fruit ripening. Types of transcriptional feedback circuits were adapted and integrated from Lü *et al.* (2018). The epigenetic tissue-specific H3K27me3 mark (red dots) represents the known changes in histone modifications from an immature to a mature fruit. (A) Model of ripening of apple fruit governed by ethylene, which acts as the primary ripening hormone. This process involves the activation of EIN3 cascade signaling in a positive feedback loop, where *MADS* genes trigger the up-regulation of the *ACC* and *ACO* genes involved in ethylene biosynthesis. However, if the process is disturbed by the ethylene competitor 1-MCP, an alternative loop is proposed. This circuit involves NAC transcription factors and auxins. (B) Model of ripening of grape fruit governed by abscisic acid, which acts as the primary ripening hormone, synthetized by the rate-limiting enzyme 9-*cis*-epoxycarotenoid dioxygenase 3 (NCED3). This process involves hormone sensing by PYR/PYL/RCAR-PP2C receptors that activated SnRK2 kinases phosphorylating and activating downstream transcription factors, such as ABF2 (ABRE-binding factor), which can in turn promote the expression of several ripening-related genes beyond MADS-box and NAC transcription factors.

Apple, as a climacteric fruit with a recent WGD, falls in the first feedback circuit, and in this fruit ripening regulation model (Fig. 3A), the ethylene transcription factor EIN3 activates expression of the MADS-box transcription factors MdMADS1 and MdMADS2, which in turn promote ethylene biosynthetic genes *ACS* and *ACO*, producing ethylene for fruit ripening in an autocatalytic system 2 circuit (Lü *et al.*, 2018). In apple leaf, this loop is silenced by the repressing epigenetic mark H3K27me3, thereby preventing ethylene production. However, unlike tomato or pear (belonging as well to the MADS feedback loop category), this epigenetic mark seems to not be present in immature fruits, while it is clearly absent in ripe ones. Downstream ripening-related genes, such as those involved in cell wall metabolism, pigment, and sugar biosynthesis, are directly linked with the ripening loop by the MADS-box transcription factor activities with the result that they are associated with ripening differentially methylated regions and tissue-specific H3K27me3.

In the presence of the ethylene competitor 1-MCP, a NAC circuit can be established, and newly synthesized or de-conjugated auxin may attempt to restore the ripening processes (Busatto *et al.*, 2021). Interestingly, these two complementary dual loops function in the same way as described for banana in the hybrid circuit, although in reverse order, opening the possibility of a more complex regulation of climacteric fruit ripening.

On the contrary, grapes, as a non-climacteric fruit, ripen under the influence of ABA. In a ripening model for grapevine (Fig. 3B) in the ABA-dependent pathway (Nicolas *et al.*, 2014), ABA binds to the PYR/PYL/RCAR–PP2C receptor complex, inactivating PP2C while activating a class III SnRK2. Once activated, SnRK2 phosphorylates the transcription factor ABF2 promoting expression of *NCED3*, whose protein constitutes a rate-limiting step in ABA biosynthesis, and a plethora of downstream ripening-related genes, such as those involved in cell wall softening and flavonoid compound synthesis. Furthermore, SnRK2 phosphorylates other transcription factors, such as members of the bZIP/ABRE, NAC, MYC/MYB, and AP2/ERF transcription factor families (Pilati *et al.*, 2017), most of which are enlisted as switch genes (Palumbo *et al.*, 2014; Massonnet *et al.*, 2017) and are highly involved in triggering ripening. In the ENCODE project, also non-climacteric species were considered, including grape, which has not undergone recent WGD and has been indicated to have homologous NAC and MADS-box transcription factors (VviNAC18 and VviSEP3, respectively), showing a gene expression pattern like climacteric species with the characteristic repressing tissue-specific epigenetic mark in leaf while being expressed in immature and ripe fruit tissues. In addition, other NAC transcription factor family members have been indicated to participate in ripening initiation, such as VviNAC03 and VviNAC60 (D’Inca *et al.*, 2023). Overall, other transcription factor families could be involved as important master regulator of the fruit ripening processes, and the continuous development in gene expression analysis technology and genome availability will enable, in the near future, their identification.

## Conclusion

Climacteric and non-climacteric fruits, such as apple and grape, are distinguished by a different regulatory mechanism governing the myriad modifications occurring in fruit to make them edible and suitable for consumption. The control of the two ripening types has been attributed to the role of two distinct hormonal signals, majorly represented by ethylene and ABA for climacteric and non-climacteric fruits, respectively. Despite this difference, recent findings have reviewed these specific controls, revealing that these hormones, together with auxin, might have a common and conserved role in coordinating the ripening processes across a range of fruits. In this review, we have shown that apple and grape, as representative fruits of the two categories, display common expression of a core set of genes involved in key ripening steps, although with different activation times. Ethylene, ABA, and auxin appear to be needed for triggering and completing the ripening mechanism in both species. Upstream of this mechanism, a key role is assigned to the action of specific classes of transcription factors, especially MADS and NAC. Although these two categories represent the ripening circuits of apple and grape, respectively, NAC elements are also proposed as possible regulators of ripening in apple, especially in the ethylene interference mechanism. These results lead to the hypothesis that while the late ripening phase might progress in a ripening-type-specific manner, the early phase might follow a common regulatory coordination. The recent identification of epigenetic markers, such as H3K27me3, may also represent a key factor in controlling autocatalytic ethylene regulation in both MADS-type and NAC-type feedback loop control. This finding provides an additional element in understanding the common regulative process across different types of ripening and suggests a conserved system regulating core ripening-related orthologs. In this scenario, climacteric and non-climacteric species might therefore have inherited from an angiosperm ancestor both genetic pathways and epigenetic regulation.

## Supplementary data

The following supplementary data are available at *JXB* online.

Fig. S1. Expression trend of apple and grapevine *NACs* belonging to *VviNAC60* clade during fruit development and ripening.

Table S1. List of genes mentioned in the text with the abbreviation, full name, and function.

Table S2. Grapevine switch genes and their expression trend during berry development and ripening.

erad324_suppl_Supplementary_Table_S1_Figure_S1

erad324_suppl_Supplementary_Table_S2
