# Supplementary material for: Molecular regulation of apple and grape ripening: exploring common and distinct transcriptional aspects of representative climacteric and non-climacteric fruits
Source: J Exp Bot. 2023 Aug 17;74(20):6207–23. doi: 10.1093/jxb/erad324 (PMC10627160; doi:10.1093/jxb/erad324)
Supplement: erad324_suppl_Supplementary_Table_S1_Figure_S1 [file erad324_suppl_supplementary_table_s1_figure_s1.pdf]

Supplementary Data

The following supplementary data are available at JXB online.

Table S1. List of genes mentioned in the text. It is provided the gene abbreviation, the full name and the broad function.

Abbreviation	Gene full name	Metabolism
3AT	Anthocyanin 3-acyltransferase	Secondary metabolism
4CL5	4-Coumarate coenzyme A ligase 5	Secondary metabolism
AAT2	Alcohol acetate transferase	Secondary metabolism
ABCG20	ABC transporter G family member 20	Transport
ABF	ABRE-binding factor	Transcription factor
ACO	1-Aminocyclopropane-1-carboxylate oxidase	Hormone metabolism - ethylene
ACS	1-Aminocyclopropane-1-carboxylate synthase	Hormone metabolism - ethylene
ANS	Anthocyanidin synthase	Secondary metabolism
AOMT	Anthocyanin O-methyltransferase	Secondary metabolism
AP2	Apetala2	Transcription factor
AREB	Abscisic acid responsive element binding protein	Transcription factor
ARF	Auxin Responsive Factor	Transcription factor
ASFT	Aliphatic suberin feruloyl transferase	Lipid metabolism
AUX/IAA	Auxin/Indole acetic acid	Hormone metabolism - auxin
BHLH	Basic helix-loop-helix	Transcription factor
CCoAOMT	Caffeoyl-coenzyme A O-methyltransferase	Secondary metabolism
CDKB	Cyclin-dependent kinase B	Cell cycle
CESA	Cellulose synthase	Cell wall
CKS1	Cyclin-dependent kinases regulatory subunit 1	Cell cycle
COBRA-like	Glycosylphosphatidylinositol anchored protein	Cell wall
CYP86B1	Cytochrome P450	Cellular metabolism / Homeostasis
EIL	Ethylene-insensitive 3-like	Transcription factor
EIL-like	Ethylene-insensitive 3-like	Transcription factor
EREBP	Ethylene-responsive element binding proteins	Transcription factor
ERF	Ethylene responsive factor	Transcription factor
ERF	Ethylene Responsive Factor	Transcription factor
ETR	Ethylene receptor	Signal transduction
EXP	Expansin	Cell wall
F3H	Flavanone-3-hydroxylase	Secondary metabolism
FAH1	Ferulic acid 5-hydroxylase 1	Secondary metabolism
FATB	Acyl-acyl carrier protein thioesterase	Lipid metabolism
GH3	Gretchen Hagen 3	Hormone metabolism - auxin
GPAT	Glycerol-3-phosphatase acyl transferase	Lipid metabolism
GPATS	Glycerol-3-Phosphate Acyltransferase 3	Lipid metabolism
GST	Glutathione S-transferase	Secondary metabolism
HD-Zip	Homeodomain-leucine zipper	Transcription factor

HDA19	Histone deacetylase 19	Histone modification
HT6	Hexose transporter 6	Sugar transporter
ILL	ILR1-like gene (IAA Leucine Resistant)	Hormone metabolism - auxin
LCYb	Lycopene β-Cyclase	Secondary metabolism
LDOX	Leucoanthocyanidin dioxygenase	Secondary metabolism
LTPG5	Non-specific lipid transfer protein GPI-anchored 5	Lipid metabolism
MADS	MADS-box	Transcription factor
MAPK	Mitogen-activated protein kinase	Signal transduction
MYB	MYB	Transcription factor
NAC	NAM, ATAF1/2, and CUC2	Transcription factor
NCED	9-cis-Epoxycarotenoid dioxygenase	Hormone metabolism - abscisic acid
NOR	Non-ripening	Transcription factor
PG	Polygalacturonase	Cell wall
PIP	Plasma membrane intrinsic protein	Aquaporins
PL	Pectate lyase	Cell wall
PP2C	Type 2C protein phosphatase	Signal transduction
PPO	Polyphenol oxidase	Secondary metabolism
PYL	Pyrabactin resistance-like	Signal transduction
PYR	Pyrabactin resistance	Signal transduction
RCAR	Regulatory component of ABA receptors	Signal transduction
RIN	Ripening inhibitor	Transcription factor
RTE	Reversion to ethylene sensitivity	Signal transduction
S6PDH	NADP-dependent D-sorbitol-6-phosphate dehydrogenase	Primary metabolism
SAM	S-adenosylmethionine synthase	Hormone ethylene
SEP	Sepallata	Transcription factor
SnRK2s	Sucrose non-fermenting 1 (SNF1)-related protein kinase 2	Response to stimuli
STS	Stilbene synthase	Secondary metabolism
SWEET10	Sugar will eventually be exported 10	Sugar transporter
TAGL	Tomato Agamous-like	Transcription factor
TIP	Tonoplast intrinsic protein	Aquaporins
TPL	Topless	Histone metabolism
UFGT	UDP glucose: flavonoid-3-O-glucosyltransferase	Secondary metabolism
WRKY	WRKY	Transcription factor
XET	Xyloglucan endotransglycosylase	Cell wall
YUC	Flavin monooxygenase-like	Hormone metabolism - auxin

Table S2. Grapevine switch genes and their expression trend during berry development and ripening. The original expression data were retrieved from Fasoli et al. (2012).

Excel file

Fig. S1. Expression trend of apple and grapevine *NACs* belonging to *VviNAC60* clade during fruit development and ripening.

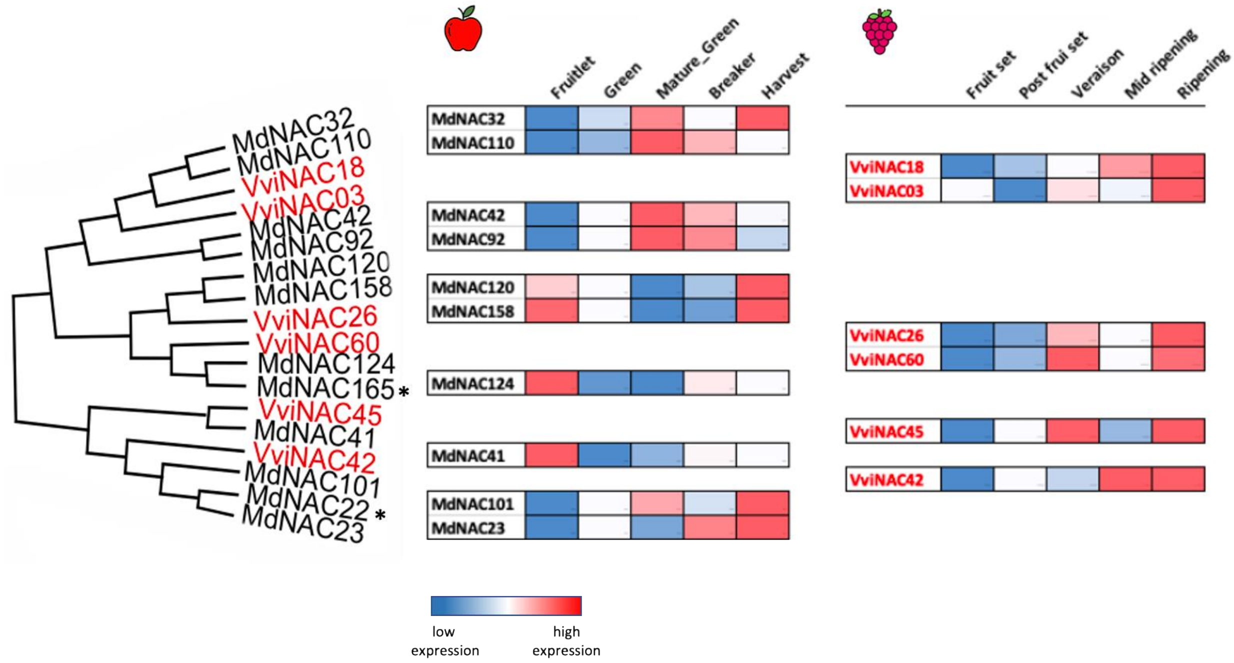

Fig. S1. Expression trend of apple and grapevine *NACs* belonging to *VviNAC60* clade during fruit development and ripening. *NACs* genes belonging to the *VviNAC60* (Fig. 2), to the left; expression trend of apple (black) and grape (red) *NACs* during five stages of fruit development and ripening, to the right. The original expression data were retrieved from Tadiello *et al.* (2016) for apple, and Fasoli *et al.* (2012) for grape. Asterisk (*) indicates a gene for which expression data are not available.
